# Acute and preventive management of anaphylaxis in German primary school and kindergarten children

**DOI:** 10.1186/s12887-015-0477-6

**Published:** 2015-10-15

**Authors:** Magdalena Kilger, Ursula Range, Christian Vogelberg

**Affiliations:** Pediatric Department, TU Dresden, University Hospital Carl Gustav Carus Dresden, Dresden, Germany; Institute for Medical Informatics and Biometry (IMB), Medical Faculty Carl Gustav Carus, Dresden, Germany

**Keywords:** Allergy, Anaphylaxis, Children, Emergency set, Kindergarten, School

## Abstract

**Background:**

Anaphylaxis is a severe, life-threatening situation. However, little is known about real-life anaphylactic management in children, especially in kindergarten and school settings, where a large number of anaphylaxes take place.

**Methods:**

Parents, school teachers and child-care providers of 86 primary schools and kindergartens in the city of Dresden, Germany, received questionnaires to report their experience with anaphylaxis in children. The main foci of interest were symptoms, allergens, sites of occurrence, acute treatment and emergency sets.

**Results:**

Out of 6352 returned questionnaires, 87 cases of anaphylaxis were identified. Prevalence was calculated at 1.5 %. Average age of the patients was 7 years, 58 % were boys. The majority of reactions occurred at home (67 %/58 children). Fourty seven percent (41 children) had recurrent episodes of anaphylaxis. Eighty two percent (71 children) showed cutaneous symptoms, 40 % (35 children) respiratory symptoms, 29 % (25 children) gastrointestinal symptoms, and 3.4 % (3 children) cardiovascular symptoms. Fourty seven percent were classified as mild reactions. Foods were the most common cause (60 %/52 cases). Out of these 52, tree-nuts (23 %/12 cases) and peanuts (16 %/8 cases) were the most frequent triggers. Sixty percent (52 cases) of reactions were treated by a physician, 35 % (30 cases) were treated by non-medical professionals only. Fifty one percent (44 children) received antihistamines, 37 % (32 children) corticosteroids, 1 % (1 child) intramuscular adrenaline. Sixty one percent of children (53 cases) received an emergency kit. Content were corticosteroids (70 %/37 cases) and antihistamines (62 %/33 cases). Adrenaline auto-injectors were prescribed to 26 % (14 cases). Concerning school and kindergarten-staff, 13 % of the child-care providers had no knowledge about the emergency kit’s content, compared to 34 % of teachers.

**Conclusions:**

This study might support the impression of severe under-treatment of anaphylactic children in the use of adrenaline and prescription of incomplete equipped emergency sets. Knowledge of school and kindergarten staff must be improved through enhanced education.

**Electronic supplementary material:**

The online version of this article (doi:10.1186/s12887-015-0477-6) contains supplementary material, which is available to authorized users.

## Background

Anaphylaxis is defined as a “severe, life-threatening generalized or systemic hypersensitivity reaction” [1, 2]. The most common causes are food, insect venom or drug allergies [3–5]. Despite studies that have shown an increasing incidence of anaphylaxis [6–8], little is known about its actual prevalence, especially in infants and children [9], and even less information exists about events within a nonmedical setting, where a large majority of reported anaphylaxes happen [9]. Furthermore, there are indications for a severe under-treatment of children with anaphylaxis, showing that 75 % of children do not receive adequate first aid [5, 10]. Deficits include both acute care as well as the prescription of emergency sets. Studies have shown that improved training of school and kindergarten staff is needed, for example in the administration of potentially life-saving medication [11–13]. The main purpose of this questionnaire-based study was to evaluate the management following an anaphylactic reaction within the kindergarten or school setting in a German metropolitan area. A point of special interest was to investigate the knowledge about the anaphylactic episodes of the afflicted children and the emergency management by parents, teachers and child-care providers. Further aspects included in the study concerned the prevalence as well as the severity of anaphylactic reactions in preschool and schoolchildren.

## Methods

### Design

In this epidemiological, cross-sectional, questionnaire-based survey, data were collected over a period of 4 months, from March 2011 until June 2011. Written consent for the study was given by both school and kindergarten authorities. Teachers, child-care providers and parents received written information about the background of the study and provided their consent by completing the questionnaires. The local ethics committee of the Technische Universität Dresden approved the study (EK67022011). The survey was completely anonymously and participation was voluntary.

### Participants

Fifty primary schools and 50 kindergartens in the city of Dresden, Germany were contacted and invited to participate in the study. To reduce possible biases, both private and public institutions were selected. Additionally, schools and kindergartens from all city districts with different social backgrounds were included in equal numbers. “Kindergarten” refers in this study to an institution that is not school-related and which is attended by children aged 1–5 years before they start primary school.

### Instrument

The questionnaires consisted of 22 items. All questions are documented in the Additional file 1 and 2. If children did not suffer from anaphylaxis, only seven questions had to be answered, whereas in the case of a child experiencing anaphylaxis, all 22 questions had to be completed. The items included the child’s age and gender, date of the first anaphylactic reaction, frequency of anaphylactic reactions, site(s) of occurrence, symptoms, causative agents, treatment including medication administered, caregiver and additional measures taken. The questions concerning the emergency kits referred to the content of the kit, the handling and the anaphylaxis emergency action plan. Additionally, parents were asked if they had informed the school’s or kindergarten’s staff about their child’s condition. Three versions of the questionnaire were designed, one for teachers, child-care providers and parents respectively. The severity of anaphylactic reactions was classified according to Muraro et al. [9]. Preceding the distribution of the questionnaires, a conventional pre-test was carried out on ten persons with a non-medical background in order to ensure the comprehensibility of the content. No problems or ambiguities were reported in the pre-test. Thereafter, schools and kindergartens were contacted personally in order to obtain a high participation rate. Questionnaires were collected after a period of 3 weeks. To increase the amount of the feedback, reminder-letters with prepaid envelopes were sent to each institution.

### Analysis

For the analyses and data processing, SPSS Version 19 for Windows® and Microsoft Excel® were used. The tests were modeled according to the Pearson’s Chi-squared test and Fisher’s exact test. Significance level was 0.05 with a 95 % confidence interval.

## Results

### Study population

Eighty six out of 100 schools and kindergarten (86 %) agreed to participate in this study. A total number of 16,644 questionnaires was distributed, out of which 6352 were completed and returned (38.2 %). Fifteen thousand three hundred eighty three questionnaires were given to parents, 654 to child-care providers, and 607 to school teachers, with a response rate of 38.7 % (*n* = 5981), 39.6 % (*n* = 259) and 18.5 % (*n* = 112) respectively.

Information provided by parents accounted for the majority of the data processed in the study. Therefore, unless otherwise stated, all data in the results section were drawn from questionnaires filled out by parents. Data obtained by teachers and child-care providers are presented separately.

### Age and gender

The average age of the 5981 children included in the study was 7 years, ranging from 12 months to 12 years. Gender was nearly equally distributed, with 2965 (49.6 %) boys and 3004 (50.2 %) girls.

### Primary anaphylactic reactions

**Fig. 1:**
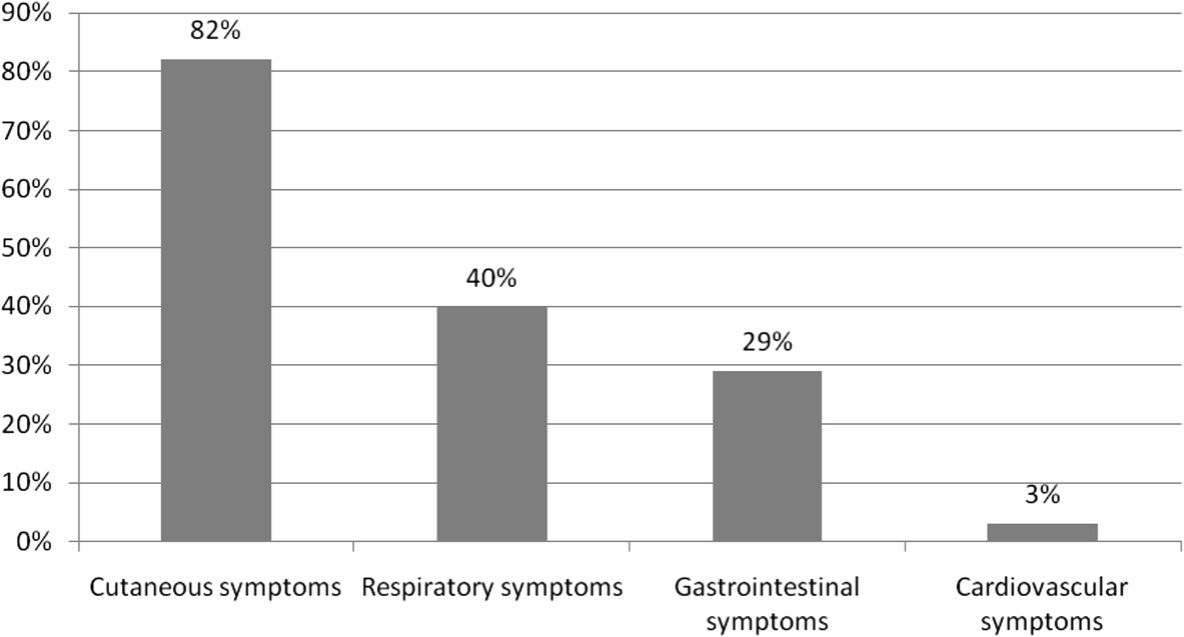
Symptoms of reported anaphylactic reactions

Eighty seven cases of anaphylaxis were reported, accounting for a prevalence rate of 1.5 %. Details on the reported cases of anaphylaxis are summarized in Table 1 and Fig. 1. In total, mild systemic reaction according to the definition of the European Academy of Allergy and Clinical Immunology (EAACI) Taskforce on anaphylaxis in children [9] accounted for 47 cases (54.0 %). Twenty eight children (32.2 %) experienced moderate systemic reactions. Three children (3.5 %) suffered a severe systemic reaction. Nine cases (10.3 %) could not be evaluated due to incomplete data.

**Table 1:** Reported cases of anaphylaxis (*n* = 87)

	Total Number	Percentage
Ratio Boys/Girls	50/37	57.5 %/42.5 %
Average age (in years) of children affected	7	
Children with a single episode of anaphylaxis	30	34.5 %
Children with 2 to 5 episodes of anaphylaxis	41	47.1 %
Children with more than 5 episodes of anaphylaxis	12	13.8 %
Missing data concerning episodes of anaphylaxis	4	4.6 %
Occurrence of the anaphylactic reaction: 6 months ago	9	10.3 %
Occurrence of the anaphylactic reaction: 12 months ago	10	11.5 %
Occurrence of the anaphylactic reaction: 18 months ago	9	10.3 %
Occurrence of the anaphylactic reaction: more than 24 months ago	56	64,4 %
Occurrence of the anaphylactic reaction: missing data	3	3.5 %
Site of occurrence of anaphylactic reaction: child’s home	58	66.7 %
Site of occurrence of anaphylactic reaction: school or kindergarten	23	26.4 %
Site of occurrence of anaphylactic reaction: relative’s/friend’s house	19	21.8 %
Site of occurrence of anaphylactic reaction: on holiday	15	17.2 %

### Triggering agents

In 88.5 % (77/87) of the described cases, the allergen responsible for the allergic reaction was identified while in 11.5 % (10/87) of the cases, the triggering allergen remained unknown. Foods were the most common cause with 59.8 % (52/87) of all reactions. Further prevalent triggers were drugs and Hymenoptera stings with 6.9 % each (6/87). The foods most frequently triggering the attacks were tree nuts (23.0 %/12 cases) and peanuts (16.1 %/8 cases), followed by hen’s egg (12.6 %/7 cases).

### Treatment

#### Profession of person giving first aid

In total, 52 out of 87 (59.8 %) cases of anaphylaxis were treated by a physician, whereas 30 cases (34.5 %) were treated by non-professionals only. In five cases (5.7 %), parents did not provide data on the person that performed first aid. From the children treated by a physician, 37.9 % (19 cases) were seen by a pediatrician, while 31.0 % (16 cases) received treatment in a hospital. Of these, 51.9 % (8 children) were admitted to the hospital and 44.4 % (7 children) were treated in outpatient care. Teachers and child-care providers reported that they only had to administer therapy in one case each (1.2 %). Parents instead performed the treatment in 43 (49.4 %) of the cases, often providing first aid before consulting a doctor additionally.

#### Medication administered

Independently of the person administering the medication, 44 (50.6 %) of the children were treated with antihistamines and/or 32 (36.8 %) with corticosteroids. Third most common was the application of inhalable β2-agonists in 17 (19.5 %) cases. Only one child (1.2 %) with an anaphylactic reaction received intramuscular adrenaline, while adrenaline by inhalation was chosen in three cases (3.5 %).

#### Emergency kits

Fifty three parents (60.9 %) reported that an emergency kit had been prescribed for their child (for details on content of emergency kits, see Table 2). The majority of them had to use their emergency set at least once, which accounts for 31 (58.5 %) cases.

**Table 2:** Content of emergency kits (*n* = 53)

	Total number	Percentage
Content of emergency kits: corticosteroids	37	69.8 %
Content of emergency kits: antihistamines	33	62.3 %
Content of emergency kits: β2-agonists	20	37.7 %
Content of emergency kits: adrenaline auto-injector	14	26.4 %

Fourty seven out of 53 parents (88.7 %) stated that they had received either theoretical or practical training in using the emergency kit. Out of the 14 children with adrenaline auto-injectors, six (35.7 %) had actually practiced how to handle the device.

**Fig. 2:**
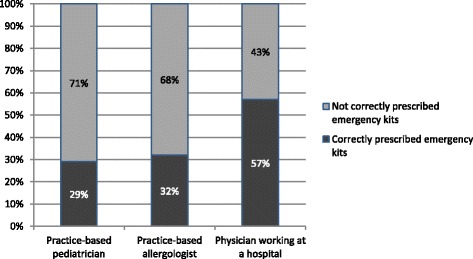
Distribution of correctly prescribed emergency kits among physicians according to their level of specialization

Practice-based pediatricians prescribed the majority of emergency sets (41.5 %), however, physicians working in a hospital were the ones who most often prescribed a correct emergency set (Fig. 2).

#### Teachers and child-care providers

**Fig. 3:**
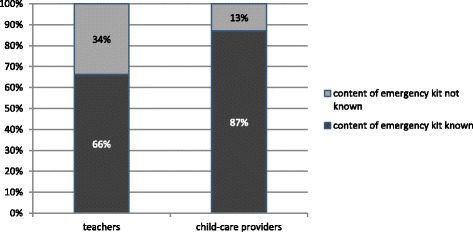
Distribution of knowledge about emergency kits content among teachers and child-care providers

Teachers and child-care providers were asked to state if they currently had a child suffering from anaphylaxis in their class/group. First of all, response rate in child-care providers was higher than in teachers (39.6 %/*n* = 259 vs. 18.4 %/*n* = 112). Child-care providers also had higher rates of reported anaphylactic reactions under their supervision (9.0 %/23 cases vs. 5.0 %/6 cases) as well as a higher rate of application of the emergency set than teachers (49.8 %/129 cases vs. 11.1 %/12 cases). Furthermore, child-care providers were more frequently informed by parents about the content as well as the correct use of the emergency set (Fig. 3).

## Discussion

This large questionnaire based study reveals two major problems in regard to the care of children with anaphylactic reactions. On one hand, there seems to be a discrepancy in the correct therapy according to current guidelines. On the other hand, parents are inadequately supplied with emergency kits and both parents and care-givers are insufficiently educated.

In accordance with other studies [3, 5], antihistamines (51 %) and corticosteroids (37 %) were the most frequently applied drugs for acute therapy. Alarmingly of the 31 moderate and severe reactions, which were treated by health professionals in 75 % of the cases, only about 5 % of the children were treated with adrenaline. This is even far less than described in comparable German studies that have shown application of adrenaline in 20 % of cases [2, 4]. It also demonstrates that almost all of the children treated by physicians most likely did not receive adequate treatment. Comparable data from another German study reports 76 % of inadequate treatment [5]. One reason for not applying adrenaline might be the physicians’ uncertainty regarding the correct diagnosis of anaphylaxis and could be improved by supporting and strengthening the diagnostic competence of physicians in general [14].

In regard to the severity of the anaphylactic reaction, the majority (54 %) of reported anaphylaxes in this study were classified as mild reactions, whereas moderate reactions accounted for 32 %. Severe reactions occurred in only 4 % of all cases. Ten percent (9/87) could not be evaluated due to lack of data. Other studies reported higher numbers of moderate and severe reactions with up to 76 % for both [5, 15]. The high number of mild anaphylactic reactions corresponds to the fact that 35 % of the parents did not seek any medical attention at all when their child had an anaphylactic reaction. Only 31 % were treated in a hospital, which is in accordance with data from the registry of German-speaking countries [3]. These facts indirectly indicate that many of the reported anaphylaxes were most likely not life-threatening but self-limiting.

Overall, the data of our study is comparable to results of other German studies, e.g. in regard to the fact that more boys than girls were affected by anaphylaxis [3, 16]. Also, the most frequent responsible allergen was food at 60 % [3, 5]. Of all foods, tree nuts (39 %) and peanuts (27 %) were the most common trigger foods, as confirmed by other studies [3, 5]. As expected [5], cutaneous symptoms (82 %) and respiratory symptoms (40 %) were the most frequently reported symptoms. However, the occurrence of respiratory, gastrointestinal (29 %) and especially cardiovascular symptoms (3 %) were considerably lower in this study. One reason for this difference might be the fact that medical laypersons participated in our study. Obviously, their competence to correctly recognize and describe symptoms is limited compared to physicians. Regarding the setting, 67 % of reactions happened at home; as confirmed by other surveys [5]. Prevalence of anaphylaxis in kindergarten and primary school children in this study is calculated at 1.5 %, which is within the range of comparable reports [17, 18].

Sixty one percent of children were prescribed an emergency kits, which is comparable to the 77 % reported in a similar study [5]. They most frequently contained antihistamines and corticosteroids. Only 26 % included an adrenaline auto-injector, which corresponds to other findings [5]. Discussions concerning the correct content of emergency kits have not reached a consensus but there are existing recommendations for Europe [9]. Interestingly, physicians seem to have different opinions on the correct prescription of emergency kits. Emergency kits were considered correctly equipped if they contained an adrenaline auto-injector, antihistamines and corticosteroids. Taking into consideration to the recommendations of Muraro et al. [9] concerning prescription of emergency medication, especially self-injectable adrenaline, only 23 % of emergency kits seemed adequately equipped. Only 36 % of the children and their families who received a prescription of an adrenaline auto-injector had been practically trained on how to use it. American studies report even less with only 17 % [19]. However, practical training is a key instrument for the correct administration of adrenaline [19], which means that an alarming lack of correct instruction and know-how exists.

The average prevalence is one child suffering from anaphylactic reactions per kindergarten or school. Surveys from the USA suggest higher rates [14, 20], whereas European rates are generally lower [21]. Slightly more child-care providers (9.0 %) than teachers (5.0 %) stated, that they had experienced a case of anaphylaxis. However, only about 1 % of teachers and about 2 % of child-care providers actually administered emergency medication. Surveys from the USA showed similar results with 3 % administered medication [22]. Fourty percent of the reactions were mild, which may explain why in 80 % of the cases, antihistamines were administered exclusively. Unlike in the USA, no teacher or child-care provider in our study has administered adrenaline [19]. In general, it seems that child-care providers have better knowledge of anaphylaxis than teachers, since they are better informed by parents.

We deliberately conducted this survey on people with no medical background, for previous studies had shown that 58 % of anaphylaxes occurred at home and up to 30 % of the cases were treated by non-health care professionals [5]. This is especially important, since children spend a considerable amount of time in school or kindergarten [5] which are consequently likely places with increased risk for anaphylaxis to occur.

Although, our study is characterized by a large number of participants, the authors are aware, that there are some relevant limitations, which should be taken into consideration and lead to a careful interpretation of the data. Despite a high effort to increase the response rate, only 39 % of the contacted persons at schools and kindergartens filled out the questionnaire. Although comparable studies showed similar response rates [23], a selection bias cannot be completely excluded. We tried to reduce a possible bias by sending the invitation to participate in the study to all districts of our city and by inviting both public and private schools and kindergartens. Furthermore, one must keep in mind that the questionnaire was answered anonymously by medical non-professionals and no medical records could be evaluated. Thus, some of the reported reactions, especially concerning mild cutaneous symptoms, might have had other reasons than anaphylaxis and the risk for false answers concerning the causing allergen for the anaphylactic reaction is higher than in studies including medical reports. Another selection bias that cannot be excluded, is the educational background of the parents, which participated in the study. In addition, it might be possible, that parents who are interested in the subject of allergic diseases preferentially participated in the study. Furthermore, the questionnaire did not include questions focusing on the reasons for the treatment decisions.

## Conclusions

In summary, the results of this large non-interventional study demonstrate that a substantial group of children with anaphylaxis does not receive adequate therapy, especially adrenaline injection according to current guidelines. Furthermore and critically, emergency kits are often not equipped correctly, especially in regard to not containing adrenaline injectors. Despite a relatively high risk for anaphylactic events to take place during the day, school and kindergarten staff is not sufficiently trained in handling children experiencing anaphylaxis. Improved guidelines based on systematic reviews [2, 9, 24] as well as a better consensus on the definition of anaphylaxis might further improve correct treatment when it occurs.

## Additional files

Additional file 1:
**Excerpt of the questionnaire for teachers/child-care providers about anaphylactic reactions in children (questions not shown concerned demographic background).** (DOCX 21 kb) Additional file 2:
**List of items that were included in questionnaire for parents about anaphylactic reactions in children.** (DOCX 26 kb)
