# Supplementary material for: Acute and preventive management of anaphylaxis in German primary school and kindergarten children
Source: BMC Pediatr. 2015 Oct 15;15:159. doi: 10.1186/s12887-015-0477-6 (PMC4608188; doi:10.1186/s12887-015-0477-6)
Supplement: Additional file 1: — Excerpt of the questionnaire for teachers/child-care providers about anaphylactic reactions in children (questions not shown concerned demographic background). (DOCX 21 kb) [file 12887_2015_477_MOESM1_ESM.docx]

Additional file 1:

Excerpt of the questionnaire for teachers/child-care providers about anaphylactic reactions in children (questions not shown concerned demographic background)

Do you know, against which allergens the child reacts?

| - Food; which ones? - Peanuts | - Soy - Egg | - Nuts - Milk |
| --- | --- | --- |
| - Wheat | - Fish / Shellfish | - Fruits |
| - Others:________________________________________________________ | | |
| - Hymenoptera venom: | - Bee | - Wasp |
| Other: ________________________________________________________ | | |
| - Drugs? Which ones?_____________________________________________ | | |

Did you inform the class mates /other children about the disease of the child?

| - Yes | - No |
| --- | --- |

Did the child experience any allergic reactions under your supervision?

| - Yes | - No |
| --- | --- |

If yes: under which circumstances?

| - During a school lesson | - During a break |
| --- | --- |
| - During a day trip | - During a school trip |
| - Others: _____________________________________________________ | |

If yes: What were the symptoms of the anaphylactic reaction? (Multiple answers are possible)

| - Headache | - Pruritus | - Redness |
| --- | --- | --- |
| - Sensation of heat | - Urticaria | - Swelling of face |
| - Prickling mouth | - Swelling of throat | - Nausea |
| - Abdominal pain | - Vomiting | - Abdominal cramps |
| - Diarrhea | - Shortness of breath | - Wheezing |
| - Coughing | - Severe dyspnea | - Apnea |
| - Vertigo | - Shivering | - Drowsiness |
| - Fainting | - Cardiac arrest | - Fear/Panic |
| - Others: _____________________________________________________________ | | |

Do you know if the child has an emergency document?

| - Yes - I don´t know | - No |
| --- | --- |

Do you know if the child has an emergency kit?

| - Yes - I don´t know. | - No |
| --- | --- |

If yes: Do you know the content of the emergency kit? (Multiple answers possible)

- Adrenalin autoinjector (e.g. Anapen™, Fastjekt™)
- Antihistamine (e.g. Fenistil™, Ceterizin)
- Corticosteroid (e.g. Prednisolone, Rectodelt™, Celestamine™, Decortine)
- Asthmaspray/ ß2 agonist (e.g. albuterol)
- Adrenalin inhalative (e.g. Infectokrupp Inhal™, Primatene™)
- I don´t know
- Others: _____________________________________________________

Was the correct use of the medication demonstrated to you?

| - Yes | - No |
| --- | --- |

If yes: Who performed the instruction?

| - Parents of the child | - The child itself |
| --- | --- |
| - Colleagues | - Others:_______________ |

Did you ever experience a situation where you had to administer the emergency medication yourself?

| - Yes | - No |
| --- | --- |

If yes: How did you treat the child? (Multiple answers possible*)*

| - Adrenaline i.m. | - Adrenaline for inhalation |
| --- | --- |
| - Corticosteroid per os | - Antihistamine per os |
| - Asthma spray | - I don´t know |

Where did you take the medication from mentioned above:

| - From the emergency kit of the child | - From a first aid kit of the school |
| --- | --- |
| - Other:__________________________________________________ | |
|  | |
