# Supplementary material for: Acute and preventive management of anaphylaxis in German primary school and kindergarten children
Source: BMC Pediatr. 2015 Oct 15;15:159. doi: 10.1186/s12887-015-0477-6 (PMC4608188; doi:10.1186/s12887-015-0477-6)
Supplement: Additional file 2: — List of items that were included in questionnaire for parents about anaphylactic reactions in children. (DOCX 26 kb) [file 12887_2015_477_MOESM2_ESM.docx]

Additional file 2:

List of items that were included in the questionnaire for parents about anaphylactic reactions in their children

1. How old is your child?

2. Sex of your child:

□ Male □ Female

3. What is the name of the school/kindergarten that your child is attending?

4. Does your child suffer from anaphylaxis (allergic shock)?

□ Yes □ No

5. Do you know whether there are other children at your child’s school/kindergarten, who are suffering from anaphylaxis?

□ Yes □ No

6. If yes: Who gave you the information about other children suffering from anaphylaxis?

□ Teacher □ Headmaster

□ Parents’ board □ Own child

□ Other parents

7. When did your child experience the first anaphylactic reaction?

About: □ 6 □ 12 □ 18 □ more than 24 months ago

8. How often did your child experience anaphylactic reactions?

□ 1x □ 2-5x □ > 5x

9. At which locations did your child experience anaphylactic reactions? (Multiple answers possible)

□ At home □ At friends/relatives □ During vacation

□ Kindergarten/School □ At the doctors practice □ At the
restaurant/cafeteria

□ At public places (on the street, cinema,…)

□ At the hospital (except for diagnostical provocations)

□ Others: ___________________________________________________

10. What were the symptoms of the first anaphylactic reaction? (Multiple answers possible)

| - Headache | - Pruritus | - Redness |
| --- | --- | --- |
| - Sensation of heat | - Urticaria | - Swelling of face |
| - Prickling mouth | - Swelling of throat | - Nausea |
| - Abdominal pain | - Vomiting | - Abdominal cramps |
| - Diarrhea | - Shortness of breath | - Wheezing |
| - Coughing | - Severe dyspnea | - Apnea |
| - Vertigo | - Shivering | - Drowsiness |
| - Fainting | - Cardiac arrest | - Fear/Panic |
| - Others: _____________________________________________________________ | | |

11. What was the cause for the anaphylactic reaction?

| - Food; which ones? - Peanuts | - Soy - Egg | - Nuts - Milk |
| --- | --- | --- |
| - Wheat | - Fish / Shellfish | - fruits |
| - Others:________________________________________________________ | | |
| - Hymenoptera venom: | - Bee | - Wasp |
| Other: ________________________________________________________ | | |
| - Drugs? Which ones?_____________________________________________ | | |

12. Who was first to treat the anaphylactic reaction?

| - Parent | - Teacher | - Child-care provider | - Emergency physician |
| --- | --- | --- | --- |
| - Family doctor | - Pediatrician | - Hospital (inpatient) | - Hospital (ICU) |
| - Hospital (outpatient) |  |  |  |

13. What kind of treatment was performed?

□ administration of emergency drugs □ emergency call (112) □ prone position

□ call on the doctor □ suppl. oxygen

□ i.v. cannula □ nothing

14. Which medication was administered?

- Adrenalin autoinjector (e.g. Anapen™, Fastjekt™)
- Adrenalin inhalative (e.g. Infectokrupp Inhal™, Primatene™)
- Antihistamine (e.g. Fenistil™, Ceterizin)
- Corticosteroid (e.g. Prednisolone, Rectodelt™, Celestamine™, Decortine)
- Asthmaspray/ ß2 agonist (e.g. albuterol)
- Others: _____________________________________________________

15. Did your child receive a so called emergency kit?

| - Yes | - No |
| --- | --- |

16. If yes: what is the content of the emergency kit?

- Adrenaline autoinjector, which one____________________________________
- Antihistamine, which one ___________________________________________
- Corticosteroide, which one___________________________________________
- Asthmaspray/ ß2 agonist, which one __________________________________
- Adrenaline inhalative, which one_____________________________________
- I don´t know
- Others: _______________________________________________________

17.Where you taught on how to use the emergency kit?

- Yes, they explained it to me
- Yes, they demonstrated the use of the kit: □ directly □ by video
- Yes, we were trained with a dummy
- No

18. Did you ever have to use an emergency kit?

□ Yes □ No

19. If you were trained, who performed the training?

| - Emergency physician | - Family doctor/pediatrician | - Allergologist |
| --- | --- | --- |
| - Pharmacist | - Hospital doctor | - Nurse |
| - Nutritionist | - Friends/family | - Patient organization |
| - I informed myself (how): ___________________________________________ | | |
| - Others: _______________________________________________________ | | |

20. Did your child receive an emergency document?

| - Yes | - No |
| --- | --- |

□ If yes, who gave it to you?________________________________

21. Does your child wear an emergency bracelet?

□ Yes □ No □ Others________________________________________

22. Did you inform the school/kindergarten about the allergy?

□ Yes, the teacher □ Yes, the director □ No, neither
